# The Rhinoplasty Rosetta Stone: Using Rasch Analysis to Create and Validate Crosswalks between the NOSE and the SCHNOS Functional Subscale

**DOI:** 10.1097/PRS.0000000000011438

**Published:** 2024-03-29

**Authors:** Floris V. W. J. van Zijl, Frank Declau, Dimitris Rizopoulos, Frank R. Datema

**Affiliations:** Rotterdam, the Netherlands; and Antwerp, Belgium; From the Departments of 1 Otorhinolaryngology and Head and Neck Surgery; 3 Biostatistics, Erasmus MC, University Medical Center Rotterdam; 2 Department of Otorhinolaryngology, GZA Hospital, Campus Sint-Vincentius

## Abstract

**Background:**

The Nasal Obstruction Symptom Evaluation (NOSE) and the Functional subscale of the Standardized Cosmesis and Health Nasal Outcomes Survey (SCHNOS-O) are widely used patient-reported outcome measures to measure functional outcomes of rhinoplasty. However, as different instruments produce scores on different metrics, results of these instruments cannot be linked directly, thus hindering comparison and aggregating of rhinoplasty outcome data from practices using either instrument. The aim of this study was to develop and validate crosswalks between the NOSE and the SCHNOS-O.

**Methods:**

In a sample of 552 rhinoplasty patients who completed both instruments, the NOSE and SCHNOS-O scales were co-calibrated onto a common interval-scaled metric using Rasch analysis. Separate Rasch models were run per instrument, and the latent constructs were estimated using the calibrated item parameters. By anchoring original patient-reported outcome measure scores of both instruments to this Rasch computed measurement scale, the scores of both instruments were linked. A second independent sample was used to validate the created crosswalks.

**Results:**

Total scores on the NOSE and SCHNOS-O were strongly correlated. The Rasch-based co-calibration of the NOSE and SCHNOS-O items resulted in a model that adequately fitted the data. Back-and-forth crosswalk tables were created from the NOSE to the SCHNOS-O. For patients with moderate nasal obstruction, predicted SCHNOS-O scores were slightly higher for a given level of the NOSE. Intraclass correlation coefficients between predicted and actual scores were 0.93 for both directions, indicating adequate agreement for group-level comparisons.

**Conclusions:**

This study developed and validated Rasch-based crosswalks from the NOSE to the SCHNOS-O and vice versa. The provided crosswalks enhance comparison and harmonization of functional rhinoplasty outcomes.

Patient-reported outcome measures (PROMs) have become the global standard to measure clinical outcomes in rhinoplasty.^1,2^ Driven by increased recognition of the importance of the patient perspective in evaluating nasal function and aesthetics, PROMs are increasingly used to quantify symptom burden, improve the shared decision-making process, and evaluate the effect of rhinoplasty.^3,4^ For the latter specifically, PROMs are expected to play a key role: the field of rhinoplasty is highly dependent on PROM data comparison, aggregation, and meta-analyses to advance its evidence levels from expert opinion and observational studies to cohort level.^5–7^ However, an important limitation of comparing and pooling outcome data are the heterogeneity of PROMs used in rhinoplasty. Outcomes from different studies or practices cannot be bridged if dissimilar PROMs are used.

Two well-established instruments for evaluating functional outcomes after rhinoplasty are the Nasal Obstruction Symptom Evaluation (NOSE) scale and the Functional subscale of the Standardized Cosmesis and Health Nasal Outcomes Survey (SCHNOS-O). Available since 2002, the NOSE is a 5-item self-report scale assessing symptoms related to nasal obstruction (obstruction, congestion, breathing, sleeping, exercise).^8,9^ The rating scale ranges from 0 to 4, with a score of 0 representing “no problem” and 4 representing “severe problem.” Containing nearly analogous components of the NOSE, The SCHNOS-O is a subscale of the SCHNOS, an instrument developed in 2017 to evaluate both functional and aesthetic rhinoplasty outcomes.^10,11^ Although both outcomes are measured when the SCHNOS is administered, the developing authors have allocated both constructs to separate subscales. The functional subscale is termed SCHNOS-O and contains 4 items related to nasal obstruction with a rating scale from 0 to 5, with 0 representing “no problem” and 5 representing “extreme problem.” Both the NOSE and SCHNOS-O are well-validated and available in multiple languages, without compelling evidence for the superiority of one over the other. The SCHNOS-O carries the advantage of an accompanied aesthetic subscale (although other aesthetic PROMs are available to accompany the NOSE in patients undergoing rhinoplasty). In contrast, surgeons that have consistently been measuring their functional outcomes for many years and, therefore, now possess large valuable data sets, have typically used the NOSE.

Although the NOSE and the SCHNOS-O include almost identical components of nasal obstruction, the wording of the items in the 2 instruments is not identical, and the number of items and rating scales differ. As different instruments produce scores on different metrics, the results of these instruments cannot be compared directly. One cannot assume, for instance, that patients scoring 50 of 100 on the NOSE experience exactly the same degree of nasal obstruction as patients scoring 50 of 100 on the SCHNOS-O. This hinders the comparison and harmonization of outcome data from practices using either instrument.

A solution to this problem could be to develop a concordance table, or crosswalk, that links scores from different PROMs that measure the same underlying construct. With such a crosswalk, scores can be converted from one instrument to another. One way to develop PROM crosswalks is using Rasch measurement techniques, a methodology that enables the projection of raw ordinal instrument scores onto a linear equal-interval scale of the latent trait that is intended to be measured.^12,13^ The scores of both instruments can be linked by anchoring the original PROM scores of both instruments to this Rasch computed measurement scale.

To the best of our knowledge, no attempt has been made to create a crosswalk between the NOSE and SCHNOS-O. Using a sample of rhinoplasty patients who completed both instruments, we therefore aim to create and validate crosswalks from the NOSE to the SCHNOS-O and from the SCHNOS-O to the NOSE.

## PATIENTS AND METHODS

### Background

To link scores between the NOSE and the SCHNOS-O, Rasch analysis was used. Rasch analysis is a type of item response theory (IRT) that has increasingly been used in psychometrics and educational testing. Compared with classic test theory, where person characteristics and test characteristics cannot be separated and thus each can be interpreted only in the context of the other, IRT enables the allocation of persons and items on a common scale independent of person and item characteristics. IRT relates the observable responses to items on a test to the unobservable latent trait that the test intends to measure. This latent trait is an aptitude dimension of a person’s ability or attribute, such as the degree of experienced nasal obstruction. The item response function posits that participants with a high degree of nasal obstruction are more likely to provide higher ratings to items than individuals with low levels of nasal obstruction.^14^ This function enables the estimation of the latent construct, the “true” degree of nasal obstruction, regardless of the specific set of items selected for the test. In other words, a respondent theoretically should exhibit the same level of nasal obstruction across different samples of test items and across various tests intended to measure nasal obstruction. By applying Rasch theory, we can consider each nasal obstruction instrument as representing different sets of test items that all measure the underlying latent trait. Determining the relative position of each (set of) test items on the latent trait scale is the principle that enables linking different instruments that measure the same construct.

**Fig. 1.**
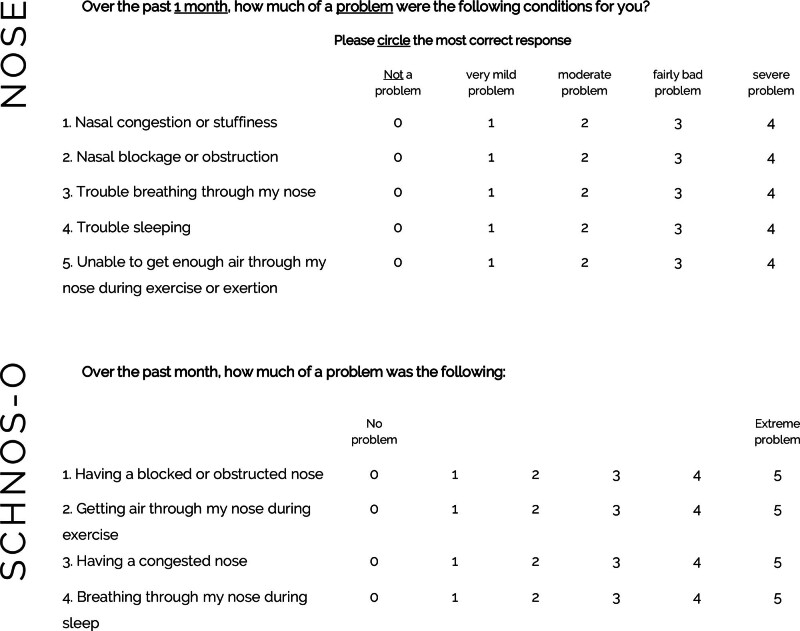
The NOSE (*above*) and the SCHNOS-O (*below*).

The NOSE and the SCHNOS-O were linked using a common person-item equating Rasch analysis. This methodology requires (1) a substantial similarity of items across both questionnaires and (2) a “linked” sample (ie, a sample of patients who have been administered both instruments).^15^ The items and rating scales of the NOSE and the SCHNOS-O are presented in Figure 1. On the item level, evident similarity between both instruments can be observed. These commonalities indicate that the instruments are representative of the same underlying construct.

### Study Setting and Participants

A sample of patients who completed both PROMs was acquired prospectively in clinical practice between September of 2021 and December of 2022 at the Department of Otorhinolaryngology and Head and Neck Surgery of the Erasmus MC, University Medical Center Rotterdam, the Netherlands, and the Department of Otorhinolaryngology, GZA Hospital, Antwerp, Belgium. The subjects were consecutive outpatients referred to explore the indication for primary or secondary rhinoplasty. The majority of patients had functional concerns or a combination of functional and aesthetic complaints. Of those undergoing rhinoplasty, almost all underwent nasal valve surgery, with or without septoplasty. The Rotterdam cohort was derived from 2 surgeons (F.V.W.J.v.Z. and F.R.D.); Antwerp patients were operated on by a single surgeon (F.D.).

The NOSE and the SCHNOS-O were administered at 3 different perioperative time points: at the first visit (preoperatively) and, if rhinoplasty was performed, 3 months postoperatively and 12 months postoperatively. We considered PROM data from both the preoperative and postoperative time points, in such a way that each unique patient figured only once in the data set. Where repeated measures were available, data were randomized to a single time point. By including both preoperative and postoperative time points, we aimed to widen the spectrum of the latent construct (nasal obstruction) examined in this study. The PROMs were either emailed to patients to complete at home or were administered by means of tablet during their routine office visit. As evidence suggests that the order of questions presented to participants affects how they respond, the order of administered PROMs was switched halfway through the study (ie, first half of total study cohort received first the NOSE and then the SCHNOS-O; the second half of included patients all received first the SCHNOS-O and then the NOSE).^16^ Two independent data sets were used for this study. The crosswalks were created using the first data set (development data set); the accuracy of the crosswalks was cross-validated using a second data set (validation data set).

### Creating the Crosswalks

Using the development data set, the NOSE and the SCHNOS-O items were first combined into 1 item bank. By performing a Rasch (1-parameter IRT) partial credit model analysis on the items of this combined item bank, step measures were created that co-calibrated the items from both scales onto a common, interval level scale (ie, logits) that measures the latent construct (ie, nasal obstruction). Then, separate Rasch models were run per instrument, to estimate the latent nasal obstruction level associated with each possible sum score. By anchoring all sum scores to the co-calibrated combined scale using the latent nasal obstruction level, each possible sum score was linked to the sum score of the other instrument for which the absolute difference in latent nasal obstruction level is smallest. This created a conversion table matching the raw scores of the NOSE to the raw scores of the SCHNOS-O, so that NOSE raw sum scores can be translated into SCHNOS-O raw sum scores and vice versa. This methodology is described in detail elsewhere.^17^

### Validation of the Crosswalks

The validity of the created crosswalks was assessed using the validation data set. Agreement between patients’ observed and crosswalked scores on the NOSE and the SCHNOS-O was assessed by computing intraclass correlation coefficients (ICCs). ICCs were considered adequate for group-level comparisons when greater than or equal to 0.70.^18^ In addition, Bland-Altman plots demonstrating the difference between predicted and observed scores were created.

This study was granted institutional review board exemption by the Medical Ethics Committees of both hospitals (Erasmus MC, MEC-2022-0532; and GZA Hospital, 190301ACADEM). All participants gave their written informed consent before enrollment and completed all parts of the questionnaire (no missing data). All raw PROM data were anonymized by removal of all patient-identifying information before transfer to the study statistician for analysis. Analyses were performed using R program v.4.2.2 (R Foundation for Statistical Computing).

## RESULTS

The development data set consisted of 552 unique rhinoplasty patients who simultaneously completed the NOSE and the SCHNOS-O at one or multiple time points. Of these, 306 were recruited in Rotterdam and 246 were recruited in Antwerp. The validation data set consisted of 100 patients: 43 from Rotterdam and 57 from Antwerp. Demographic characteristics were comparable between both samples (Table 1). Mean NOSE and SCHNOS-O scores were slightly higher in the development sample, which is explained by a higher percentage of preoperative time points in this data set.

**Table 1.** Patient Characteristics

	Development Sample	Validation Sample
No.	552	100
Sex, % female	56.7	55.0
Mean age ± SD, yr	33.6 ± 13.7	3.9 ± 13.0
Center, % Antwerp	44.6	57.0
Time point, % preoperatively	63.9	50.0
Mean NOSE ± SD	50.1 ± 32.4	42.6 ± 32.2
Mean SCHNOS-O ± SD	54.0 ± 33.2	47.7 ± 33.1

**Fig. 2.**
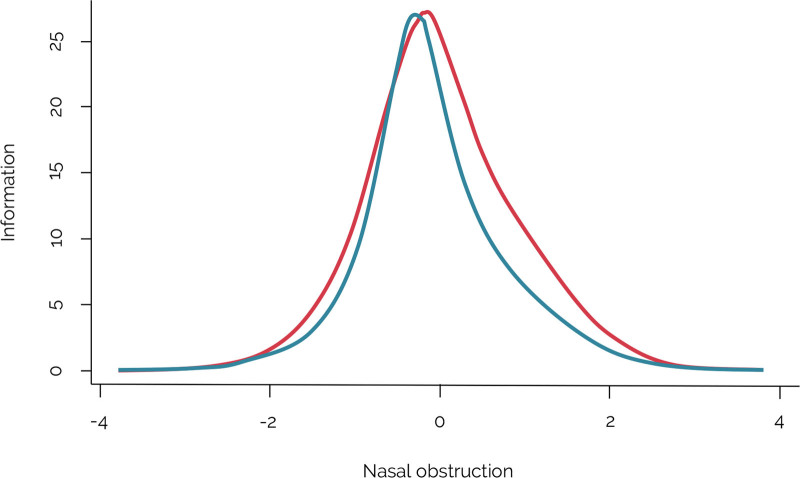
Test information function curves of the NOSE (*red*) and the SCHNOS-O (*blue*). The test information function is the sum of all separate item information functions. Higher positive theta scores indicate worse nasal obstruction.

NOSE and SCHNOS-O scores were highly correlated (Spearman *r* = 0.92; *P* < 0.001). The Rasch-based co-calibration of the NOSE and SCHNOS-O resulted in a model that adequately fitted the data. Descriptive and model summary data are provided. (**See Document, Supplemental Digital Content 1**, which shows descriptives and model summary, http://links.lww.com/PRS/H64.) All items demonstrated good model fit, with mean square infit ranging from 0.57 to 1.08 and outfit ranging from 0.60 to 1.26. Person reliability (Cronbach α) of the co-calibrated scale was 0.96. The test information curves of the NOSE and SCHNOS-O are plotted in Figure 2. These curves demonstrate the reliability of each PROM in measuring certain levels of nasal obstruction. Both scales measured a comparable range of nasal obstruction, with high precision in measuring moderate levels of nasal obstruction, and less precision in measuring the extreme ends of nasal obstruction levels.

Table 2 presents the Rasch-based crosswalks between the NOSE and SCHNOS-O. Raw scores of both instruments are linked through the latent trait level generated from each Rasch analysis, anchored at the co-calibrated scale. For the NOSE, trait levels varied from −1.59 logits (raw sum score, 0) to 1.78 logits (raw sum score, 100). Trait levels computed from the SCHNOS-O ranged from −1.52 logits (raw sum score, 0) to 1.64 logits (raw sum score, 100). For each NOSE sum score, the latent trait level of the SCHNOS-O sum score that was closest to the latent trait level of that particular NOSE sum score was selected. For instance, the nasal obstruction level corresponding to a NOSE raw score of 50 (−0.04 logits) was closest to the nasal obstruction level corresponding to a SCHNOS-O raw score of 55 (−0.08 logits).

**Table 2.** Crosswalks from NOSE to SCHNOS-O and from SCHNOS-O to NOSE

Crosswalk		Crosswalk	
NOSE to SCHNOS-O		SCHNOS-O to NOSE	
0	0	0	0
5	5	5	5
10	10	10	10
15	15	15	15
20	20	20	20
25	30	25	25
30	35	30	25
35	40	35	30
40	45	40	35
45	50	45	40
50	55	50	45
55	60	55	50
60	65	60	50
65	70	65	55
70	75	70	65
75	80	75	70
80	85	80	75
85	90	85	80
90	95	90	85
95	95	95	90
100	100	100	100

### Cross-Validation of Results

**Fig. 3.**
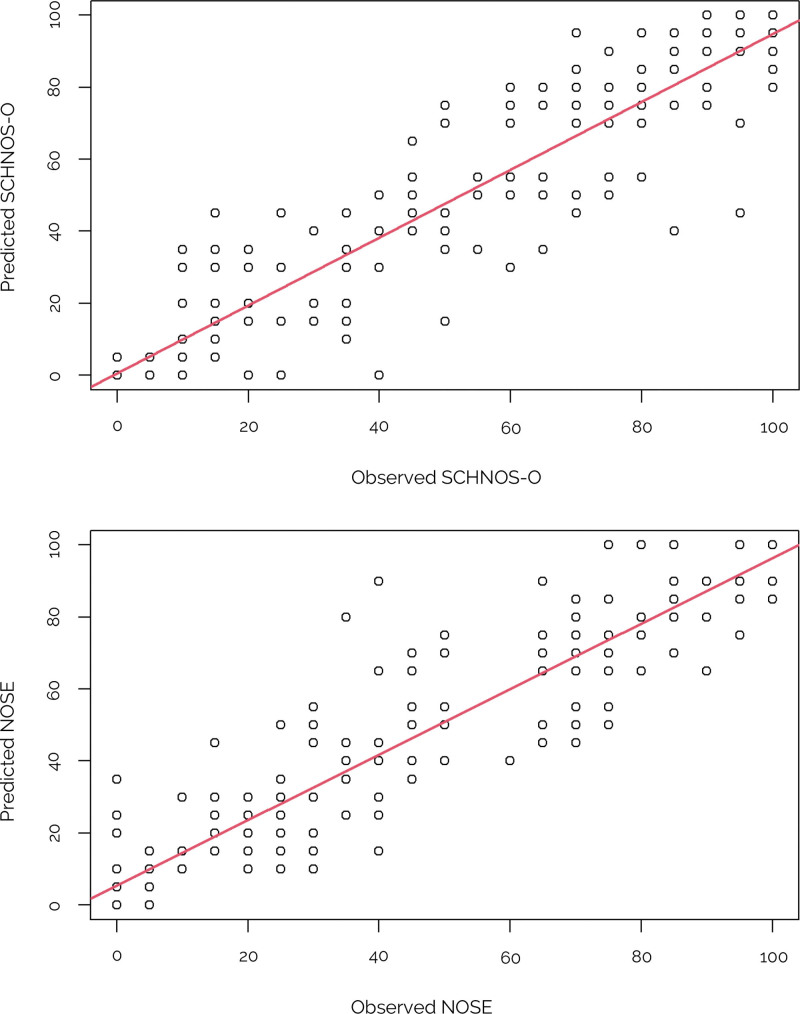
(*Above*) Correlation between predicted and observed scores of the SCHNOS-O. (*Below*) Correlation between predicted and observed scores of the NOSE.

**Fig. 4.**
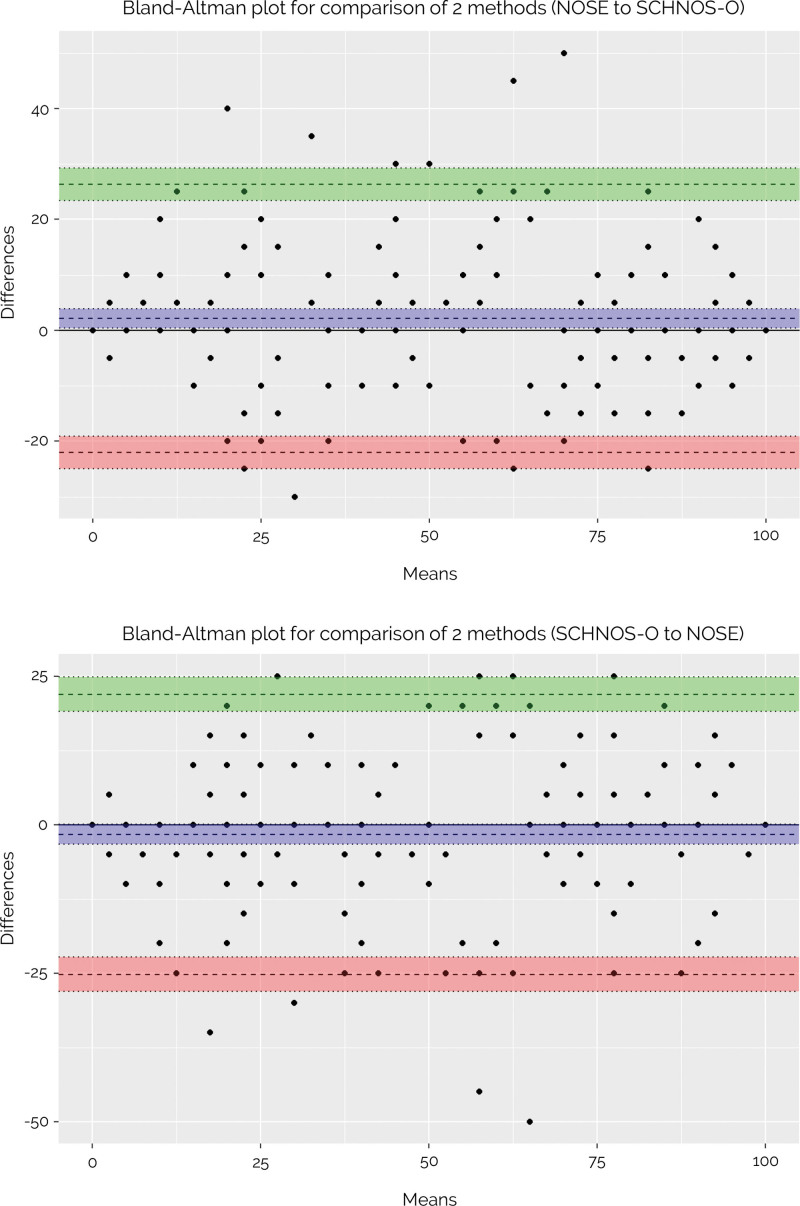
(*Above*) Bland-Altman plot demonstrating agreement between observed and predicted scores (NOSE to SCHNOS-O). The *y* axes depict the difference between observed and predicted scores; the *x* axes represent the mean of observed and predicted scores. The *dashed line in blue* represents the mean difference between both scores. The *dashed lines in green* and *red* demonstrate the 95% limits of agreement. (*Below*) Bland-Altman plot demonstrating agreement between observed and predicted scores (SCHNOS-O to NOSE). The *y* axes depict the difference between observed and predicted scores; the *x* axes represent the mean of observed and predicted scores. The *dashed line in blue* represents the mean difference between both scores. The *dashed lines in green* and *red* demonstrate the 95% limits of agreement.

A high degree of agreement between observed scores and scores predicted from the crosswalks was found (Fig. 3). For both scales, ICCs between crosswalked and observed scores were 0.93 (95% CI, 0.91 to 0.95), indicating excellent agreement for group-level comparisons. Figure 4 demonstrates Bland-Altman plots of intraindividual differences between observed and predicted scores (Fig. 4). Substantial discrepancies in agreement between individual patients were noted. In 4 participants, these discrepancies exceeded the limits of agreement. These findings conclude that these crosswalks are suitable for use at the group level, but not at the individual patient level.

## DISCUSSION

In this study, Rasch analysis was used to link 2 widely used scales for measuring patient-reported nasal obstruction in rhinoplasty, the NOSE and the SCHNOS-O. The presented crosswalks enable the rhinoplasty surgeon to convert NOSE scores to SCHNOS-O scores and vice versa. The application of the crosswalks in an independent sample of patients undergoing rhinoplasty indicated that the crosswalks can be validly used for group-level analyses in rhinoplasty populations.

Linking established rhinoplasty PROMs enhances meaningful comparison and interpretation of rhinoplasty outcomes. Although the NOSE and SCHNOS-O are linguistically much alike and only slight differences between raw and crosswalked scores were observed in this study, using a validated linking method is the only way to permit data conversion between these two instruments.^19^ The ability to pool NOSE and SCHNOS-O data enables the sharing of data, comparing results across studies and populations, creating large cohorts or registries for collaboration in multicenter studies, and facilitating the pooling of data in systematic reviews and meta-analyses. Because the results of Rasch analysis are sample and test independent, these crosswalks can be used for all past and future NOSE-to–SCHNOS-O or SCHNOS-O–to-NOSE score conversions. To use the crosswalks provided by this study, surgeons can transform each original sum score in their data set to the crosswalked scores per Table 2 using common statistical analytic software.

Ideally, global consensus on the use of a single outcome instrument in rhinoplasty practices would obviate the need for crosswalks. A single assessment tool clearly provides the best solution for seamless monitoring of functional rhinoplasty outcomes. In various health care areas, standardized instruments are appointed through core outcome sets (COS). In a COS, the best available instrument to measure a particular outcome in a particular disease or population is selected by relevant stakeholders (ie, patients, physicians, and policymakers) based on factors such as relevance, feasibility, and measurement properties.^20^ In rhinoplasty, however, the adoption of a common instrument could potentially meet resistance. Most rhinoplasty PROMs have been developed by large research groups with significant support from geographically or socially affiliated surgeons and practices. It will be difficult to persuade such groups to abandon the PROM they developed themselves, which has often been a time-consuming process, and convert to using another. This is particularly relevant if the PROM suggested by the COS is not intrinsically better than the one already used, as is the case with the NOSE and the SCHNOS-O.^21^ Second, surgeons who have established prospective registries using a particular instrument might be reluctant to switch to a different instrument. If historical PROM data cannot be bridged with a new PROM, this would imply a loss of historical data. With the availability of crosswalks, surgeons can continue using their PROM of choice, as long as it has evidence of adequate quality, while preserving the value of previously collected data. With the results of this study, for instance, surgeons measuring their outcomes using the SCHNOS can continue using both subscales of the SCHNOS, but are now able to translate data from the functional subscale to outcome data derived from the NOSE (and vice versa for NOSE users).

A strength of the current study is the use of IRT. Although other methodologies to link scores from instruments that measure similar constructs exist, IRT offers a flexible and powerful framework for score linking by its inherent ability to calibrate different items of the same construct on a common underlying metric.^22,23^ Alternative crosswalk methods available in the literature, such as linear equating, assume scores are linearly related and do not account for measurement error, which could result in biased mappings.^24^ Other strengths of this study are the relatively large sample size and the fact that all participants completed both scales in their entirety, which enhances robust score linking.^15^

A relevant issue is the breadth of applicability of these crosswalks in other language versions of the NOSE and the SCHNOS. Cross-cultural use of these crosswalks depends on whether the construct is indeed similar when measured in culturally or linguistically different groups. For the Dutch NOSE and SCHNOS versions used for these crosswalks, construct equivalence has primarily been established through the Dutch validation studies of these PROMs, designed to qualitatively maximize construct equivalence using international guidelines.^9,11^ These validation studies also reported statistical validity and reliability measurements that were similar to the source-language PROMs. Intercultural variability can further be investigated using differential item functioning (DIF). Declau et al. found no substantial DIF of the NOSE within gender or ethnicity.^25^ For the SCHNOS, no DIF evaluation has been performed, so strictly speaking, further studies in this field are necessary. Yet, it is likely that the SCHNOS-O performs similarly stable transculturally, as it is nearly analogous to the NOSE. We therefore conclude that although transcultural construct equivalence can never be guaranteed, it is reasonable to assume that these crosswalks are suitable for use across different validated language versions of the NOSE and the SCHNOS.

Although common with PROM crosswalks, a limitation of this study is that the validity of the crosswalks could only be established for cohort-level conversion.^26^ The ICCs between observed and predicted scores were adequate for group-level analyses, but the Bland-Altman plots indicated that individual crosswalked scores were not sufficiently accurate for individual-level recoding. This implies that rhinoplasty surgeons cannot use these crosswalks to convert scores in individual patients. However, individual score conversion was not the primary goal of developing these crosswalks; the assets of cohort-level pooling or comparisons carry much greater potential value than individual score translations.

## CONCLUSIONS

This study developed and validated crosswalks from the NOSE to the SCHNOS-O and vice versa. The Rasch co-calibrated crosswalk tables enable rhinoplasty surgeons and researchers to convert nasal obstruction data across both instruments. The implications of translating scores across rhinoplasty outcome instruments are profound: comparable data facilitate the evaluation and aggregation of large data sets, which are crucial to address clinical knowledge gaps and raise evidence levels in a time of need.

## DISCLOSURE

The authors declare no potential conflicts of interest with respect to the research, authorship, and publication of this article. The authors received no financial support for the research, authorship, and publication of this article.

## Supplementary Material


