# Supplementary material for: The Rhinoplasty Rosetta Stone: Using Rasch Analysis to Create and Validate Crosswalks between the NOSE and the SCHNOS Functional Subscale
Source: Plast Reconstr Surg. 2024 Mar 29;154(5):955–63. doi: 10.1097/PRS.0000000000011438 (PMC11512607; doi:10.1097/PRS.0000000000011438)
Supplement: Supplementary file 1 [file prs-154-00955-s001.pdf]

Supplement 1. Descriptives and model summary.

Descriptives

```
##
## Descriptive statistics for the 'DF_NOSE[-length(DF_NOSE)]' data-set
##
## Sample:
##  5 items and 552 sample units; 0 missing values
##
## Proportions for each level of response:
##           0-Geen probleem 1-Licht probleem 2-Matig probleem
## NLNOSE1_1          0.1576           0.1793           0.1848
## NLNOSE1_2          0.2536           0.1612           0.1793
## NLNOSE1_3          0.1884           0.1703           0.1630
## NLNOSE1_4          0.3514           0.1214           0.1504
## NLNOSE1_5          0.2428           0.1395           0.1341
##           3-Redelijk ernstig probleem 4-Ernstig probleem
## NLNOSE1_1                      0.3043             0.1739
## NLNOSE1_2                      0.2609             0.1449
## NLNOSE1_3                      0.2609             0.2174
## NLNOSE1_4                      0.1993             0.1775
## NLNOSE1_5                      0.2428             0.2409
##
##
## Frequencies of total scores:
##       5  6  7  8  9 10 11 12 13 14 15 16 17 18 19 20 21 22 23 24 25
## Freq 45 27 36 25 20 23 19 17 22 15 22 20 24 30 28 37 30 28 29 26 29
##
##
## Cronbach's alpha:
##                        value
## All Items             0.9372
## Excluding NLNOSE1_1 0.9259
## Excluding NLNOSE1_2 0.9200
## Excluding NLNOSE1_3 0.9095
```

```
## Excluding NLNOSE1_4 0.9316
## Excluding NLNOSE1_5 0.9266
##
##
## Pairwise Associations:
##    Item i Item j p.value
## 1       1      5  <2e-16
## 2       2      4  <2e-16
## 3       1      4  <2e-16
## 4       4      5  <2e-16
## 5       2      5  <2e-16
## 6       3      4  <2e-16
## 7       1      3  <2e-16
## 8       2      3  <2e-16
## 9       3      5  <2e-16
## 10      1      2  <2e-16
```

```
##
## Descriptive statistics for the 'DF_SCHNOSF[-length(DF_SCHNOSF)]' data-set
##
## Sample:
##  4 items and 552 sample units; 0 missing values
##
## Proportions for each level of response:
##          Geen probleem 0      1      2      3      4 Extreem proleem 5
## SCHNOS_1          0.2065 0.1196 0.0906 0.1739 0.2409            0.1685
## SCHNOS_2          0.2120 0.1159 0.0815 0.1612 0.2246            0.2047
## SCHNOS_3          0.1431 0.1413 0.1105 0.1504 0.2772            0.1775
## SCHNOS_4          0.2464 0.1087 0.0688 0.1250 0.2083            0.2428
##
##
## Frequencies of total scores:
##       4  5  6  7  8 9 10 11 12 13 14 15 16 17 18 19 20 21 22 23 24
## Freq 42 29 28 23 33 7 15 11 14 12 12 29 30 25 34 27 42 35 40 23 41
```

```
##
##
## Cronbach's alpha:
##                     value
## All Items          0.9292
## Excluding SCHNOS_1 0.9023
## Excluding SCHNOS_2 0.9078
## Excluding SCHNOS_3 0.9067
## Excluding SCHNOS_4 0.9147
##
##
## Pairwise Associations:
##   Item i Item j p.value
## 1      1      2   0.001
## 2      1      4   0.001
## 3      2      3   0.001
## 4      2      4   0.001
## 5      3      4   0.001
## 6      1      3  <2e-16
```

Model summary

```
##
## Call:
## gpcm(data = data_IRT, constraint = "1PL")
##
## Coefficients:
## $NLNOSE1_1
## Catgr.1  Catgr.2  Catgr.3  Catgr.4   Dscrmn
##  -1.117   -0.391   -0.024    1.201    1.960
##
## $NLNOSE1_2
## Catgr.1  Catgr.2  Catgr.3  Catgr.4   Dscrmn
##  -0.560   -0.201    0.206    1.333    1.960
```

```
##
## $NLNOSE1_3
## Catgr.1  Catgr.2  Catgr.3  Catgr.4   Dscrmn
##  -0.919   -0.306   -0.017    0.944    1.960
##
## $NLNOSE1_4
## Catgr.1  Catgr.2  Catgr.3  Catgr.4   Dscrmn
##  -0.049   -0.111    0.317    1.070    1.960
##
## $NLNOSE1_5
## Catgr.1  Catgr.2  Catgr.3  Catgr.4   Dscrmn
##  -0.574   -0.241   -0.073    0.823    1.960
##
## $SCHNOS_1
## Catgr.1  Catgr.2  Catgr.3  Catgr.4  Catgr.5   Dscrmn
##  -0.722   -0.329   -0.377    0.233    1.171    1.960
##
## $SCHNOS_2
## Catgr.1  Catgr.2  Catgr.3  Catgr.4  Catgr.5   Dscrmn
##  -0.686   -0.297   -0.414    0.183    0.963    1.960
##
## $SCHNOS_3
## Catgr.1  Catgr.2  Catgr.3  Catgr.4  Catgr.5   Dscrmn
##  -1.161   -0.485   -0.305    0.011    1.164    1.960
##
## $SCHNOS_4
## Catgr.1  Catgr.2  Catgr.3  Catgr.4  Catgr.5   Dscrmn
##  -0.495   -0.175   -0.345    0.064    0.773    1.960
##
##
## Log.Lik: -5831.281
```

```
Itemfit Statistics:
            Chisq  df p-value Outfit MSQ Infit MSQ Outfit t Infit t Discrim
```

NLNOSE1_1	449.319	500	0.949	0.897	0.892	-1.624	-1.730	0.823
NLNOSE1_2	393.203	500	1.000	0.785	0.832	-3.147	-2.679	0.846
NLNOSE1_3	299.199	500	1.000	0.597	0.566	-6.726	-7.879	0.902
NLNOSE1_4	631.464	500	0.000	1.260	1.084	2.410	1.201	0.791
NLNOSE1_5	472.427	500	0.807	0.943	0.932	-0.693	-0.983	0.846
SCHNOS_1	415.860	500	0.997	0.830	0.890	-2.397	-1.610	0.866
SCHNOS_2	509.708	500	0.372	1.017	0.957	0.247	-0.594	0.861
SCHNOS_3	462.108	500	0.887	0.922	0.959	-1.140	-0.591	0.839
SCHNOS_4	633.498	500	0.000	1.264	1.057	2.769	0.772	0.838
